# ¿La función muscular inspiratoria podría ser un equivalente de la insuflación pulmonar en los pacientes con EPOC?

**DOI:** 10.1016/j.opresp.2021.100084

**Published:** 2021-02-20

**Authors:** Mariela Alvarado Miranda, Cinta Cumplí Gargallo, Antonio Sancho Muñoz, Mireia Admetlló Papiol, Juana Martínez Llorens, Ana Balañá Corberó

**Affiliations:** a Servei de Pneumologia-URMAR, Hospital del Mar (IMIM), Barcelona, España; b CEXS, Universitat Pompeu Fabra, Barcelona, España

**Keywords:** EPOC, Biomarcadores, Obstrucción bronquial, Hiperinsuflación pulmonar, Disfunción muscular inspiratoria, COPD, Biomarkers, Bronchial obstruction, Lung hyperinflation, Inspiratory muscle dysfunction

## Abstract

**Introducción:**

La enfermedad pulmonar obstructiva crónica (EPOC) es la patología respiratoria que causa mayor morbimortalidad a nivel mundial. Los parámetros de función pulmonar y las manifestaciones sistémicas se han definido como factores pronósticos, sin embargo, presentan limitaciones. El objetivo ha sido analizar si la fuerza muscular inspiratoria sería un reflejo de la insuflación pulmonar y, por tanto, un factor pronóstico de los pacientes con EPOC.

**Método:**

Se seleccionaron pacientes con EPOC que se realizaron previamente una valoración de la fuerza muscular respiratoria no invasiva y una prueba de función pulmonar desde enero de 2015 hasta octubre de 2017. Posteriormente, se revisó la mortalidad hasta el 1 de marzo de 2020.

**Resultados:**

Se incluyeron 140 pacientes con EPOC (estadio GOLD I 5%, II 73,4% y III 21,6%) de los cuales un 10% fallecieron durante el seguimiento. La obstrucción bronquial, definida por volumen espiratorio forzado en el primer segundo (FEV_1_) fue un buen predictor de mortalidad (p = 0,004). La hiperinsuflación pulmonar, definida como relación capacidad inspiratoria/capacidad pulmonar total (CI/CPT) inferior a 25 y CI inferior al 65% de los valores de referencia, incrementaba la mortalidad en los pacientes con EPOC (p = 0,001 y p = 0,06, respectivamente). En la presente cohorte la fuerza de los músculos inspiratorios, valorada mediante la presión nasal durante una inhalación máxima (SNIP) no fue un factor pronóstico (p = 0,629).

**Conclusión:**

En los pacientes con EPOC, la hiperinsuflación pulmonar es un factor pronóstico, no así la función muscular inspiratoria. La función muscular inspiratoria de los pacientes de los EPOC no solo depende de la mecánica pulmonar, sino que también de factores intrínsecos de los propios músculos.

## Abstract (en)

**Introduction:**

Chronic obstructive pulmonary disease (COPD) is the respiratory disease that causes the greatest morbidity and mortality worldwide. Lung function parameters and systemic manifestations have been defined as prognostic factors; however, they have limitations. The aim of this study was to analyze whether inspiratory muscle strength could reflect lung hyperinflation, and therefore serve as a prognostic factor in COPD patients.

**Method:**

We selected COPD patients who had performed a non-invasive respiratory muscle strength assessment and lung function testing between January 2015 and October 2017. Mortality was subsequently followed up until March 1, 2020.

**Results:**

We included 140 COPD patients (GOLD stage I 5%, II 73.4%, and III 21.6%), of whom 10% died during follow-up. Bronchial obstruction, defined by FEV_1_, was a good predictor of mortality (p = 0.004). Lung hyperinflation, defined as inspiratory capacity (IC)/total lung capacity less than 25 and IC less than 65% of predicted increased mortality in COPD patients (p = 0.001 and p = 0.06, respectively). In this cohort, inspiratory muscle strength, measured by SNIP, was not a prognostic factor (p = 0.629).

**Conclusion:**

In COPD patients, lung hyperinflation is a prognostic factor, but inspiratory muscle function is not. Inspiratory muscle function in COPD patients depends not only on lung mechanics but also on intrinsic muscle factors.

## Introducción

La enfermedad pulmonar obstructiva crónica (EPOC) se caracteriza por síntomas respiratorios persistentes asociado a una limitación progresiva y poco reversible del flujo aéreo^1^. A pesar de ser una enfermedad respiratoria, los pacientes presentan síntomas extrapulmonares, denominados manifestaciones sistémicas^2^. La EPOC, actualmente es la enfermedad respiratoria que causa mayor morbimortalidad a nivel mundial^3^ y se estima que en el año 2030 será la cuarta causa de muerte^4^. La variabilidad entre los pacientes con EPOC ha hecho difícil definir biomarcadores, que nos ayuden en el pronóstico de estos enfermos^5^. Actualmente, tanto los síntomas, los parámetros de función pulmonar y muscular, así como las manifestaciones sistémicas, han demostrado que podrían ser biomarcadores en la EPOC^6^, ^7^, ^8^, ^9^, ^10^, ^11^, ^12^, ^13^, ^14^, ^15^, ^16^, ^17^. Dentro de los síntomas, el grado de disnea ha demostrado que puede ser un factor pronóstico^6^, ^7^, aunque limitado por la subjetividad. Diversos parámetros de la función pulmonar también se han estudiado. Clásicamente, el grado de obstrucción bronquial, expresado por el volumen espiratorio forzado en el primer segundo (FEV_1_), es un factor pronóstico, aunque fundamentalmente solo para pacientes con estadios graves^8^, ^9^, ^10^, ^11^. La hiperinsuflación pulmonar en la EPOC, medida como la relación capacidad inspiratoria/capacidad pulmonar total (CI/CPT) inferior a 25, también se ha descrito como un factor independiente de mortalidad^12^ e incluso mejor que la obstrucción bronquial^13^ para los pacientes con EPOC. La valoración de la hiperinsuflación pulmonar no es accesible para todos los pacientes ya que se precisa la medición de los volúmenes pulmonares estáticos. Esto ha hecho que se estudien otras variables que están relacionadas con la hiperinsuflación pulmonar y que son más sencillas de medir en la práctica clínica habitual. La función muscular inspiratoria analizaría de manera indirecta la insuflación pulmonar. En concreto en los pacientes con EPOC, la disfunción muscular inspiratoria sería secundaria a la presencia de hiperinsuflación pulmonar^18^ y esta a su vez, dependería de la obstrucción bronquial y por tanto a la pérdida de elasticidad pulmonar^19^. Moore et al. demostraron que la fuerza muscular inspiratoria es un biomarcador del pronóstico en los pacientes con EPOC^17^. Sin embargo, otros autores han demostrado que los músculos inspiratorios de los pacientes con EPOC, y fundamentalmente el diafragma, se producen cambios adaptativos con el objetivo de mejorar la fuerza de los mismos^20^. En una reciente revisión sobre biomarcadores en la EPOC se indica que se debería de avanzar en el estudio de la relación entre la hiperinsuflación pulmonar y la función muscular inspiratoria debido a los estudios con resultados contradictorios^5^. El objetivo de este trabajo es analizar si la disfunción muscular inspiratoria es un predictor de mortalidad en una cohorte de pacientes con EPOC y, por eso podría sustituir a la valoración de la hiperinsuflación pulmonar en estos enfermos.

## Material y métodos

### Población y diseño del estudio

Estudio retrospectivo que analiza la mortalidad de los pacientes con EPOC en función de la fuerza inspiratoria y la insuflación pulmonar. Se incluyeron todos los pacientes con EPOC en los que se había valorado la función muscular respiratoria desde enero de 2015 hasta octubre de 2017 en nuestro laboratorio. Ha sido realizado de acuerdo con las guías para la investigación con humanos de la *World Medical Association*^21^ y con la aprobación del comité de Ética de nuestro centro, el que consideró que no era necesario la obtención del consentimiento informado.

Los pacientes incluidos fueron adultos de raza caucásica, con diagnóstico de EPOC según los criterios GOLD^1^. Se excluyeron aquellos enfermos con problemas para la comprensión y/o realización de las exploraciones, clínica de obstrucción nasal, otras patologías respiratorias, síntomas o signos de compromiso de los músculos respiratorios y ausencia de seguimiento en la historia clínica de atención primaria o hospitalaria superior a tres meses.

### Técnicas

#### Pruebas de función pulmonar

A todos los pacientes se les realizó una espirometría forzada (*EasyOne*, ndd Medical Technologies, Zurich, Suiza) basal, así como otra tras la administración de broncodilatador, de acuerdo con las normativas existentes^22^. Posteriormente, se procedió a la determinación de los volúmenes pulmonares estáticos y de la resistencia de las vías aéreas por pletismografía corporal (Masterlab, Jaeger, Würzburg, Alemania) según normativas^23^. También se midió la capacidad de transferencia de monóxido de carbono (DLCO) con el medidor de gases incorporado en el citado equipo, según la técnica de «respiración única»^24^. Para todos los valores de referencia se los valores publicados para la población mediterránea. Se realizó la clasificación de la gravedad de la EPOC según los estadios de GOLD valorando solamente el grado de obstrucción bronquial^1^.

#### Pruebas de fuerza muscular respiratoria

La valoración de la función muscular respiratoria había sido solicitada por sus neumólogos habituales. Se determinaron las presiones estáticas máximas en boca, generadas tanto durante el esfuerzo inspiratorio, presión inspiratoria máxima medida en boca (PIM), presión espiratoria máxima medida en boca (PEM), según las normativas actuales^25^, ^26^. Se utilizó una pieza bucal ocluible (SIBEL, Barcelona, España) conectada a un manómetro de presión (TSD 104, Biopac Systems, Goleta, CA. U.S.A.), cuya señal se registró mediante un polígrafo digital (Biopac Systems). La maniobra de PIM se realizó mediante una inspiración máxima desde volumen residual (RV), mientras que para la PEM se efectuó una espiración máxima desde CPT. Se incluyó en el análisis el valor máximo obtenido en tres maniobras aceptables y con una variación inferior al 20%, expresándose los valores como relativos a los de referencia para población mediterránea^27^. Se realizó también la determinación de la presión nasal durante una inhalación máxima (SNIP), con el sujeto en sedestación y tras la oclusión de un orificio nasal por un catéter de presión conectado al mismo polígrafo digital^25^, ^26^, ^28^. El sujeto hizo un mínimo de 10 maniobras de inhalación forzada desde capacidad funcional residual (CRF) seleccionando el mejor valor^25^, ^26^, ^28^. SNIP inferior a 70 cmH_2_O en hombres y a 60 cmH_2_O en mujeres indica disfunción muscular inspiratoria^25^.

### Mortalidad

Se solicitó al Departamento de Salud de nuestra Comunidad Autónoma, la mortalidad de estos pacientes hasta el 1 de marzo de 2020. El seguimiento varió entre 25 y 63 meses.

### Análisis estadístico

Las variables cuantitativas de distribución normal se presentan como media y desviación típica (x ± SD), menos las cualitativas que se expresa como frecuencia. La comparación de las variables cuantitativas, que tenían una distribución normal, se realizó mediante la prueba paramétrica *t* de Student y las cualitativas con la prueba no paramétrica *χ*^*2*^.

El modelo de regresión no paramétrico de Cox se ha usado para la valoración de las variables de función pulmonar y fuerza muscular respiratoria que tenían efecto sobre la mortalidad. Se representan las curvas *Receiver Operating Characteristic* (ROC) y el área bajo la curva (AUC) ha sido usado para comparar como de poderosa es cada una de las variables para predecir mortalidad. Finalmente, la curva de supervivencia de Kaplan-Meier para todas las causas de fallecimiento ha sido realizada para los factores pronósticos encontrados.

Los datos fueron analizados con el programa informático de tratamiento de datos SPSS versión 17 (*SPSS Statistics for Windows*, Version 25.0. IBM Corp. Armonk, New York, USA), fijándose la significancia estadística como p ≤ 0,05.

## Resultados

Desde enero de 2015 hasta octubre de 2017, se realizaron un total de 692 valoraciones la fuerza muscular respiratoria. De estos, 196 eran pacientes con EPOC. Cuarenta y dos de estos pacientes no realizaron correctamente la maniobra y en nueve de ellos no se obtuvieron datos sobre la supervivencia. Finalmente, se han incluido 140 pacientes con EPOC, divididos en los estadios de gravedad GOLD I 5%, II 73,4% y III 21,6%.

Durante el periodo de seguimiento, los pacientes fallecidos fueron 14 (10% del total), los cuales tenían más edad, mayor obstrucción bronquial, mayor atrapamiento aéreo e hiperinsuflación pulmonar y un menor intercambio de gases pulmonares que los supervivientes. Entre ambos grupos de pacientes no encontramos diferencias en la fuerza de los músculos inspiratorios (tabla 1).

**Tabla 1:** Características generales, antropométricas y de función pulmonar de la población

	Vivos (n = 126)	Fallecidos (n = 14)	p
Edad, años	64 (8)	69 (8)	0,032
Hombres/ Mujeres	77 H / 49 M	11 H / 3 M	0,199
IMC, kg/m^2^	25,3 (5,2)	25,1 (6,0)	0,870
%FEV_1_ /FVC	0,47 (0,13)	0,38 (0,11)	0,018
CVF, %ref	81 (18)	63 (17)	0,005
FEV_1_, %ref	49 (18)	33 (11)	0,000
CRF, %ref	150 (34)	159 (39)	0,153
CPT, %ref	115 (19)	108 (16)	0,153
VR, %ref	191 (53)	194 (46)	0,804
% VR/CPT	59,1 (10,0)	65,1 (7,4)	0,006
CI, %ref	76 (24)	55 (19)	0,001
%CI/CPT	31,6 (9,8)	24,6 (7,8)	0,007
DLCO, %ref	51 (18)	41 (25)	0,141
KCO, %ref	53 (17)	41 (18)	0,083
PIM, cmH_2_O	72 (22)	67 (12)	0,295
PEM, cmH_2_O	132 (42)	117 (37)	0,197
SNIP, cmH_2_O	70 (21)	67 (14)	0,525

H: hombre; M: mujer; IMC: índice de masa corporal; %FEV_1_/FVC: cociente entre el volumen espiratorio forzado en el primer segundo y la capacidad vital forzada; CVF: capacidad vital forzada; %ref; expresados en relación con los valores de referencia; FEV_1_: volumen espiratorio forzado en el primer segundo; CPT: capacidad pulmonar total; VR volumen residual; %RV/CPT: relación entre volumen residual y capacidad pulmonar total; CRF capacidad residual funcional; CI: capacidad inspiratoria; %CI/CPT: relación entre capacidad inspiratoria y capacidad pulmonar total; DLCO: capacidad de transferencia de monóxido de carbono; KCO: capacidad de transferencia de monóxido de carbono corregido por el volumen alveolar; PIM: presión inspiratoria máxima medida en boca; PEM: presión espiratoria máxima medida en boca; SNIP: presión nasal durante una inhalación máxima.

Las variables se expresan como media y desviación típica, (estadística realizada mediante *t* de Student) a excepción del género que son número (test estadísticos *χ*^*2*^).

**Figura 1:**
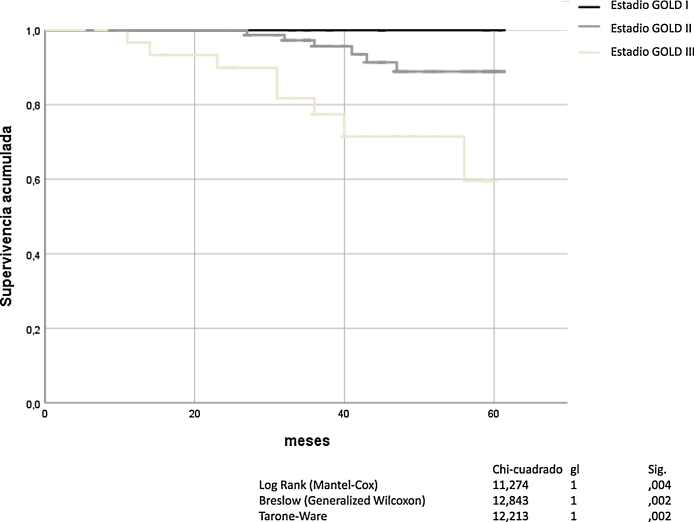
Mortalidad de los pacientes con EPOC en función de los estadios GOLD (definidos como obstrucción bronquial).

La obstrucción bronquial, definida por FEV_1_ y graduada con la escala de GOLD, fue un buen predictor de mortalidad por todas las causas (Mantel-Cox *χ*^*2*^ 11,274, p = 0,004; Breslow *χ*^*2*^ 12.843, p = 0,02; Tarone-Ware χ^2^ 12.213, p = 0,002). El análisis de Kaplan-Meier para todas las causas de muerte en función de la gravedad de la obstrucción bronquial según GOLD, se muestra en la figura 1.

**Figura 2:**
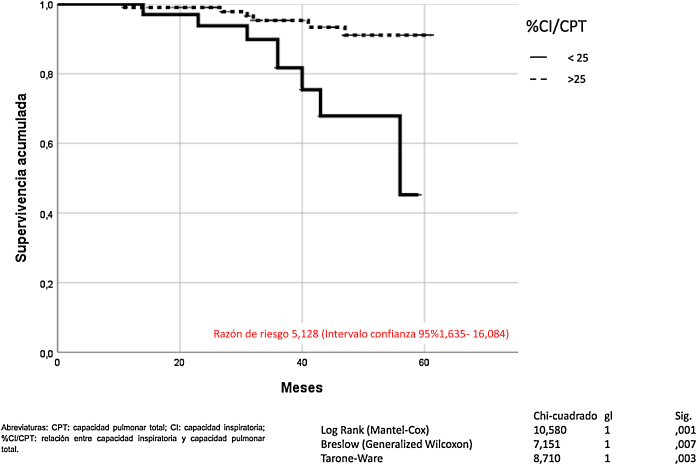
Mortalidad de los pacientes con EPOC en función de la hiperinsuflación pulmonar definida por la variable CI/CPT.

**Figura 3:**
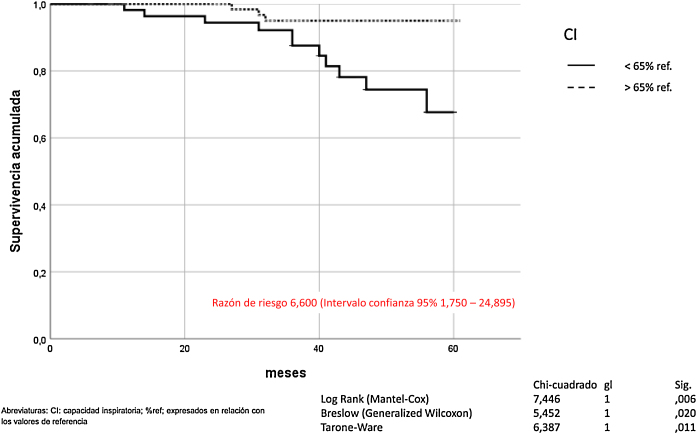
Mortalidad de los pacientes con EPOC en función de la hiperinsuflación pulmonar definida por la variable CI.

La hiperinsuflación pulmonar, definida como CI/CPT inferior a 25, incrementaba la mortalidad por todas las causas en los pacientes con EPOC (Mantel-Cox *χ*^*2*^ 10.580, p = 0,001; Breslow *χ*^*2*^ 7.151, p = 0,007; Tarone-Ware *χ*^*2*^ 8.710, p = 0,003) (fig. 2). La CI inferior a 65% de los valores de referencia, correspondía a una relación CI/CPT menor de 25. La mortalidad por todas las causas de los pacientes con EPOC que presentan CI inferior al 65% fue superior de manera estadísticamente significativa (Mantel-Cox *χ*^*2*^ 7.446, p = 0,006; Breslow *χ*^*2*^ 5.452, p = 0,020; Tarone-Ware χ^2^ 6.387, p = 0,011) (fig. 3).

**Figura 4:**
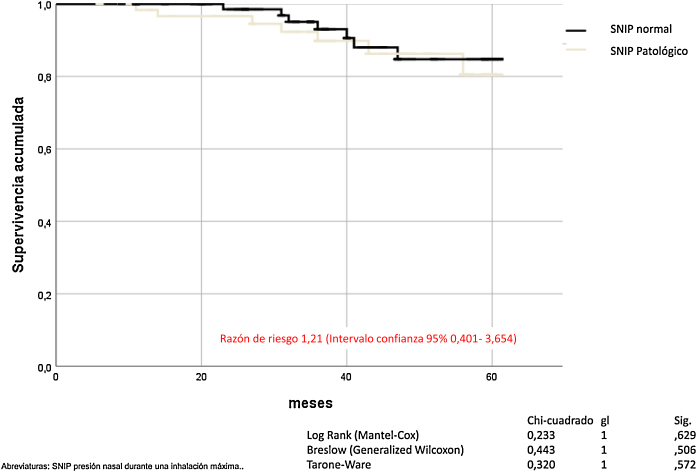
Mortalidad de los pacientes con EPOC en función de la fuerza muscular inspiratoria.

La SNIP no influye en la mortalidad por todas las causas de los pacientes con EPOC (Mantel-Cox *χ*^*2*^ 0,233, p = 0,629; Breslow *χ*^*2*^ 0,443, p = 0,506; Tarone-Ware *χ*^*2*^ 0,320, p = 0,572) (fig. 4).

**Figura 5:**
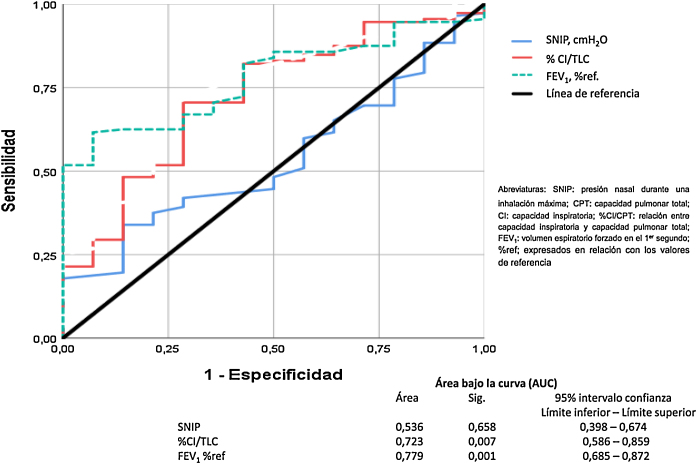
Análisis de la curva ROC para comparar el FEV1, %CI/CPT, y la SNIP en los pacientes con EPOC.

Finalmente, el análisis de la curva ROC para comparar el FEV_1_, la relación CI/CPT y la SNIP está representado en la figura 5. Estos datos demuestran que la obstrucción bronquial y la hiperinsuflación pulmonar son buenos predictores de mortalidad por todas las causas en nuestros pacientes con EPOC. Sin embargo, la SNIP no es un buen discriminador para predecir la mortalidad por todas las causas de nuestros pacientes.

## Discusión

Con este estudio se ha confirmado que la mortalidad de los pacientes con EPOC se asocia directamente con la hiperinsuflación pulmonar e inversamente con la obstrucción bronquial y que la disfunción muscular inspiratoria no es un factor pronóstico.

Un biomarcador es aquella característica objetiva y medible que evalúa un proceso biológico, tanto normal como patológico, así como la respuesta a los tratamientos^29^. Existen multitud de trabajos con el objetivo de encontrar un biomarcador en la EPOC, valorando fundamentalmente la capacidad predictora de mortalidad^5^, para ello se han analizado los parámetros de función pulmonar y muscular, los síntomas respiratorios y también las manifestaciones sistémicas asociadas a la enfermedad^5^, ^6^, ^7^, ^8^, ^9^, ^10^, ^11^, ^12^, ^13^, ^14^, ^15^, ^16^, ^17^.

De forma concordante con nuestros resultados, en los pacientes con EPOC la obstrucción bronquial es un predictor de mortalidad^6^, ^7^, ^8^, ^9^, aunque tiene limitaciones. Por este motivo algunos autores han asociado al grado de obstrucción bronquial diferentes manifestaciones sistémicas, generando los índices multidimensionales, con la finalidad de mejorar su capacidad predictiva^1^, ^30^, ^31^. Sin embargo, para la realización de estos índices es necesario tanto pruebas de función pulmonar como valoraciones clínicas o nutricionales o de la capacidad de esfuerzo, dificultando así su aplicación en la práctica clínica habitual. Esto ha hecho que se sigan estudiando otros parámetros de función pulmonar como biomarcadores en la EPOC. Dentro de estos, la hiperinsuflación pulmonar, al igual que en nuestro trabajo, ya había demostrado ser un factor de mal pronóstico en la EPOC^13^, ^17^. En los pacientes con EPOC, la obstrucción bronquial produce atrapamiento aéreo progresivo que se traduce en un aumento en la capacidad residual funcional y finalmente acaba con una disminución de la CI, y en consecuencia, en hiperinsuflación pulmonar^19^. En nuestro estudio hemos valorado la hiperinsuflación pulmonar mediante una relación CI/CPT inferior a 25^13^. Por consiguiente, para la valoración de la insuflación pulmonar se precisa una espirometría lenta para medición de la CI y una pletismografía corporal o dilución de gases para medir la CPT^22^, ^32^, ^33^. Esto hace que sea una valoración compleja técnicamente, ya que precisa de la realización de dos tipos de pruebas de función pulmonar. Es por esto que se han propuesto pruebas indirectas para la valoración de la insuflación pulmonar en los pacientes con EPOC. Moore et al. publicaron que la disfunción muscular inspiratoria era un factor pronóstico para los pacientes con EPOC, ya que reflejaba la hiperinsuflación pulmonar^17^. La hiperinsuflación pulmonar produce un aplanamiento del diafragma con pérdida de la longitud óptima para la contracción muscular, disminuyendo la fuerza ejercida por este, es decir provoca disfunción muscular inspiratoria^18^. Sin embargo, este razonamiento fisiopatológico no es del todo acertado porque en los músculos inspiratorios de los pacientes con EPOC, concretamente en el diafragma, se producen cambios adaptativos para vencer la posición desfavorable y generar más fuerza^20^. Es por eso que actualmente la presencia de disfunción muscular inspiratoria en los pacientes con EPOC está en debate, así como su utilidad como factor pronóstico^5^. Los resultados de nuestro trabajo demuestran que la disfunción muscular inspiratoria no es un factor pronóstico, y por tanto avalaría los resultados de la adaptación de los músculos respiratorios de estos enfermos. Los resultados diferentes entre este trabajo y el de Moore et al.^17^ pueden ser explicados por las diferencias metodológicas. En ambos trabajos la función muscular inspiratoria solo se valoró mediante la SNIP. La SNIP es la determinación de la fuerza de los músculos inspiratorios en la nariz durante una inhalación forzada^25^. En el trabajo de Moore et al. se realizó la clasificación de los pacientes con EPOC en función de la media y desviación estándar de la SNIP y no según la definición de disfunción muscular inspiratoria^17^. Los valores normales de la SNIP se consideran mayor de 60 cmH_2_O para mujeres y 70 cmH_2_0 para hombres, es por esto que cifras inferiores definen disfunción muscular inspiratoria^25^. En nuestro trabajo sí que hemos valorado realmente la presencia de disfunción muscular inspiratoria. Esta variación en la definición de la disfunción muscular posiblemente pueda explicar esta diferencia en los resultados.

En este trabajo también hemos demostrado que la CI solamente también es un factor pronóstico para los pacientes con EPOC. Los enfermos que tienen una CI inferior al 65% de los valores de referencia presentan una mayor mortalidad. La CI refleja de una manera indirecta la insuflación pulmonar en los pacientes con EPOC^19^. La medición de la CI es técnicamente más sencilla, ya que solo precisa de una espirometría lenta^22^.

Las limitaciones del presente trabajo se derivan por ser un estudio retrospectivo. Las valoraciones fueron solicitadas por su equipo médico habitual, normalmente para otros trabajos de investigación del grupo. Con el fin de evitar un sesgo, se incluyeron a todos los pacientes con EPOC que se habían valorado en nuestro laboratorio, salvo que hubiese una sospecha clínica de enfermedad neuromuscular. Las valoraciones de la función pulmonar y de la fuerza muscular respiratoria se realizaron de acuerdo con las normativas vigentes, y por tanto, no existen diferencias entre las exploraciones realizadas a los pacientes. Los autores no llevaron a cabo ninguna intervención en los pacientes durante el periodo de seguimiento, y cada uno siguió los tratamientos indicados por su neumólogo. Aún así, los pacientes eran tratados por neumólogos de nuestro servicio y ninguno de ellos fue incluido en ensayos clínicos. La causa del fallecimiento, así como las comorbilidades, se han recogido desde la historia clínica de los pacientes. Todas estas limitaciones afectan por igual a todos los pacientes, por lo que no invalidaría nuestros resultados.

Una limitación metodológica es que no hemos medido la presión esofágica durante una maniobra de inspiración máxima (Pesofsniff). La Pesofsniff es la mejor forma para medir la fuerza de los músculos inspiratorios^25^. Sin embargo, diversos trabajos ya han demostrado que existe una buena correlación entre la SNIP y la Pesofsniff en los pacientes con EPOC^25^, ^34^, ^35^, ^36^, ^37^. En nuestro trabajo también realizamos la medición de la fuerza muscular inspiratoria en boca con la PIM en los pacientes. Empero, no hemos utilizado la PIM para la valoración de la fuerza de los músculos inspiratorios por tener peor correlación con la P_esof_sniff en los pacientes con EPOC^25^, ^34^, ^35^, ^36^, ^37^.

En resumen, en los pacientes con EPOC son factores determinantes del pronóstico la obstrucción bronquial y la hiperinsuflación pulmonar. La hiperinsuflación pulmonar valorada tanto mediante la determinación de los volúmenes pulmonares estáticos como solo mediante la evaluación de la CI. Nuestros resultados demuestran que la fuerza muscular inspiratoria no es un factor pronóstico en los pacientes con EPOC. La hiperinsuflación pulmonar no puede ser valorada mediante la fuerza muscular inspiratoria, ya que existen factores adaptativos de los propios músculos inspiratorios.

## Conflicto de intereses

Los autores declaran no tener ningún conflicto de intereses.
